# Preferred Migration of Mitochondria toward Cells and Tissues with Mitochondrial Damage

**DOI:** 10.3390/ijms232415734

**Published:** 2022-12-12

**Authors:** Seo-Eun Lee, Young Cheol Kang, Yujin Kim, Soomin Kim, Shin-Hye Yu, Jong Hyeok Park, In-Hyeon Kim, Hyeon-Young Kim, Kyuboem Han, Hong Kyu Lee, Sung-Hwan Kim, Chun-Hyung Kim

**Affiliations:** 1 Paean Biotechnology Inc., 5 Samil-Daero 8-gil, Jung-gu, Seoul 04552, Republic of Korea; 2 Jeonbuk Branch Korea Institute of Toxicology, Jeongeup 56212, Republic of Korea

**Keywords:** mitochondria, transplantation, trafficking, mitochondrial dysfunction

## Abstract

Mitochondria are organelles that play a vital role in cellular survival by supplying ATP and metabolic substrates via oxidative phosphorylation and the Krebs cycle. Hence, mitochondrial dysfunction contributes to many human diseases, including metabolic syndromes, neurodegenerative diseases, cancer, and aging. Mitochondrial transfer between cells has been shown to occur naturally, and mitochondrial transplantation is beneficial for treating mitochondrial dysfunction. In this study, the migration of mitochondria was tracked in vitro and in vivo using mitochondria conjugated with green fluorescent protein (MT^GFP^). When MT^GFP^ were used in a coculture model, they were selectively internalized into lung fibroblasts, and this selectivity depended on the mitochondrial functional states of the receiving fibroblasts. Compared with MT^GFP^ injected intravenously into normal mice, MT^GFP^ injected into bleomycin-induced idiopathic pulmonary fibrosis model mice localized more abundantly in the lung tissue, indicating that mitochondrial homing to injured tissue occurred. This study shows for the first time that exogenous mitochondria are preferentially trafficked to cells and tissues in which mitochondria are damaged, which has implications for the delivery of therapeutic agents to injured or diseased sites.

## 1. Introduction

Mitochondria play a fundamental role in cellular survival and growth by supplying energy in the form of ATP via oxidative phosphorylation. In addition to producing ATP, mitochondria are involved in various physiological processes, including cellular metabolism, apoptosis, inflammation, innate immunity, and calcium homeostasis. Given their critical roles in cellular physiology, mitochondrial dysfunction is involved in many human diseases, including metabolic syndromes, inflammatory diseases, neurodegeneration, cancer, and aging [1,2,3]. Mitochondrial dysfunction has also been linked to mitochondrial DNA mutation, excessive mitochondrial reactive oxygen species (ROS) production, reduced ATP generation, aberrant calcium homeostasis, and impaired mitochondrial biogenesis [4].

Isolated mitochondria can be transferred to any cell type via simple coincubation or brief centrifugation in vitro [5,6,7,8]. Isolated mitochondria can also be internalized into tissues through local or systemic injection in vivo [9,10]. It was suggested that mitochondrial internalization would be mediated by micropinocytosis [11,12] or actin-dependent endocytosis [13]. In animal models and patients, the injection of autologous or nonautologous mitochondria has been effective in treating injury and diseases, including ischemia/reperfusion injury, spinal cord injury, mouse fatty liver, cognitive deficits, inflammatory diseases, and Parkinson’s disease [14,15,16,17,18,19,20,21]. Although the underlying mechanisms of such effects are not fully understood, it has been suggested that the transfer of healthy mitochondria (or mitochondrial transplantation) ameliorates mitochondrial defects and helps recover cellular function by increasing mitochondrial biogenesis or replacing abnormal mitochondria with healthy mitochondria [22,23,24,25]. Recently, our data have demonstrated that mitochondrial transplantation attenuated the lipopolysaccharide-induced inflammation in vitro and in vivo by blockade of the phosphorylation, nuclear translocation, and trans-activity of NFκB [26]. Therefore, the transfer of fully functional mitochondria into defective cells or tissues could be an effective therapeutic strategy for treating mitochondrial dysfunction.

The key to successful mitochondrial transplantation therapy is the trafficking of mitochondria to the target cells, in which they can exert their biological effects. Therefore, systemically administrated mitochondria must have abilities that guide them to the sites with mitochondrial damage. Mesenchymal stem cells (MSCs) have been used in cell therapy because they have the capacity to home to target tissues and engraft into these tissues appropriately. Several studies have shown that systemically injected autologous and allogenic MSCs preferentially migrate to and promote the functional recovery of injured tissue [27,28,29,30,31,32]. Thus, intravenously injected mitochondria might also localize preferentially to damaged tissues; however, unlike the well-established MSC homing process, mitochondrial trafficking to the sites of injury is not well studied.

In the present study, we isolated mitochondria conjugated with green fluorescent protein (MT^GFP^) from stable HEK293 cells expressing translocase of the outer membrane 20 (TOM20) fused to an upstream green fluorescent protein (GFP). In a coculture system, MT^GFP^ was internalized in a cell type-specific manner. We also found that selective MT^GFP^ transplantation depended on the mitochondrial function of the receiving fibroblasts. Furthermore, compared with MT^GFP^ injected intravenously into normal mice, MT^GFP^ injected intravenously into bleomycin (BLM)-induced idiopathic pulmonary fibrosis (IPF) mice located more abundantly in the lung tissue, suggesting that mitochondrial trafficking to damaged cells and tissues occurred.

## 2. Results and Discussion

### 2.1. Transplantation of MT^GFP^ into Human Lung Fibroblasts

To obtain mitochondria that express GFP consistently, human TOM20 was fused at the N-terminus of the GFP to generate the plasmid pCMV_TOM20_GFP. The expression of TOM20_GFP was confirmed in HEK293 cells using a western blotting assay with an anti-GFP antibody (Figure S1a). To observe the localization of TOM20_GFP, HEK293 cells were stained with MitoTracker Red CMXRos, followed by a primary antibody against an anti-GFP antibody and an Alexa Fluor 488-conjugated secondary antibody. An overlap was detected in MitoTracker Red CMXRos and GFP staining, suggesting that GFP was localized in the mitochondria (Figure S1b). We also prepared mitochondrial, and cytosol fractions from HEK293 cells transfected with pCMV_TOM20_GFP and analyzed their GFP expression via a western blotting assay, wherein the COX IV and β-tubulin proteins were used as mitochondrial and cytosol markers, respectively. The immunostaining of GFP was detected in the mitochondria but not in the cytosol, indicating that GFP was exclusively localized in the mitochondria (Figure S1c). Mitochondria conjugated with GFP were referred to as the MT^GFP^.

**Figure 1:**
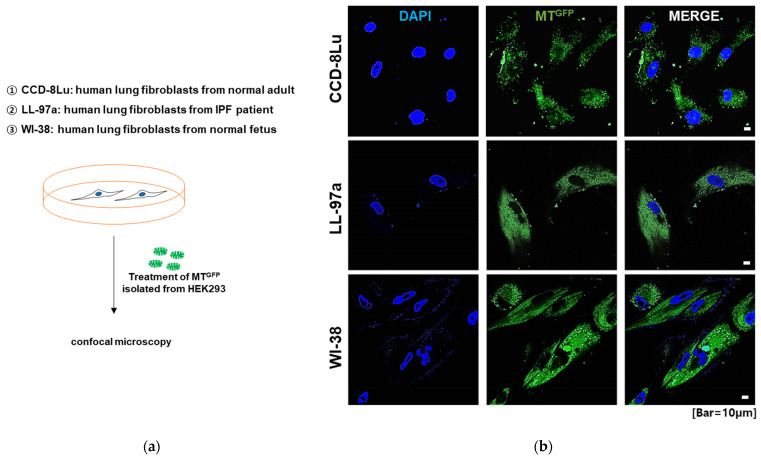
Transplantation of MTGFP into human lung fibroblasts. (**a**) Schematic diagram of human fibroblast monoculture and MTGFP treatment. (**b**) Representative immunofluorescence images of each fibroblast. Cell nuclei were stained with DAPI. MTGFP were successfully transplanted into human lung fibroblasts, including the human lung fibroblast CCD-8Lu, the human lung diploid fibroblast WI-38, and the idiopathic pulmonary fibrosis patient-derived human lung fibroblast LL97A. Scale bar: 10 μm.

To test whether MT^GFP^ could be transferred to various human lung fibroblasts, including the human lung fibroblasts CCD-8Lu, the human lung diploid fibroblasts WI-38, and the IPF patient-derived human lung fibroblasts LL97a, we isolated MT^GFP^ from HEK293 cells stably expressing TOM20_GFP using differential centrifugation and then treated them to each fibroblast. As shown in Figure 1, when isolated MT^GFP^ were simply coincubated with each fibroblast, they were successfully transplanted into each type of fibroblast. 

Various methods can be used for mitochondrial transplantation into cells, e.g., coincubation [33,34], a cell-penetrating peptide [35,36], MitoCeption [8,37], and Mitopunch [38]. Although coincubation is limited by the endocytosis effect of recipient cells, it is easy to perform and useful for examining mitochondrial transfer. Our data showed that the internalization of isolated MT^GFP^ into recipient fibroblasts by coincubation was confirmed.

### 2.2. Selective Transplantation of MT^GFP^ into Human Lung Fibroblasts in a Coculture System

**Figure 2:**
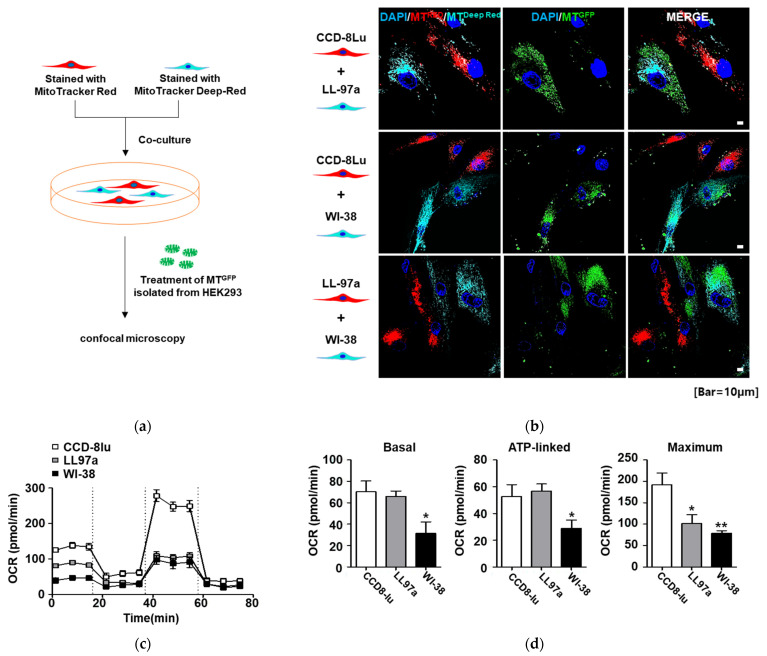
Selective transplantation of MTGFP into human lung fibroblasts in a coculture system. (**a**) Coculture model of three types of human lung fibroblast. Each fibroblast type was prestained with MitoTracker Red CMXRos or MitoTracker Deep-Red, as described in the Materials and Methods section. An equal density of each stained cell type was coplated, treated with MTGFP, and then imaged via a confocal microscope. (**b**) Selective transplantation of MTGFP in the coculture system. LL-97a or WI-38 fibroblasts prestained with MitoTracker Deep-Red took up much more MTGFP than was taken up by CCD-8Lu fibroblasts prelabeled with MitoTracker Red CMXRos (top and middle panels). Fluorescent images revealed that MTGFP were more abundant in WI-38 fibroblasts (deep red) than in LL97a fibroblasts (red) (low panel). Nuclear staining is shown in blue (DAPI). Scale bars: 10 μm. (**c**) Bioenergetics profiles of three human lung fibroblast types. Representative OCRs were measured in CCD-8Lu, LL97a, and WI-38 fibroblasts. OCR values under basal conditions were recorded and analyzed. Subsequently, 1 µM oligomycin, 1 µM FCCP, and 0.5 µM rotenone/antimycin A were injected into the medium (1 × 10^4^ cells/well). Representative data are shown from three independent experiments. (**d**) Basal, ATP-linked, and maximal OCR levels. Basal OCR was measured before oligomycin treatment, and maximum OCR was measured after FCCP treatment and subtraction of the nonmitochondrial-derived OCR measurement following rotenone and antimycin A treatment. All OCR values were normalized using the cell mass measured with methylene blue (* *p* < 0.05 and ** *p* < 0.01).

To determine whether mitochondria differentially transfer into cells, we used a direct coculture system. First, CCD-8Lu fibroblasts were prestained with MitoTracker Red CMXRos, and LL97a fibroblasts were prelabeled with MitoTracker Deep-Red. Subsequently, CCD-8Lu (red) and LL97a (deep red) fibroblasts were cocultured in the same well and treated with isolated MT^GFP^ for 6 h, after which the cells were observed using confocal microscopy (Figure 2a). Surprisingly, more isolated MT^GFP^ were found in LL97a fibroblasts than in CCD-8Lu fibroblasts, suggesting that mitochondria might be preferentially transplanted to specific cell types (Figure 2b). To exclude the effect of MitoTracker dye, CCD-8Lu fibroblasts prestained with MitoTracker Deep-Red and LL97a fibroblasts prelabeled with MitoTracker Red CMXRos were cocultured and treated with MT^GFP^ (Figure S2a). Regardless of MitoTracker dye, more isolated MT^GFP^ were transplanted to LL97a fibroblasts than to CCD-8Lu fibroblasts (Figure S2b). Additionally, in cocultures of WI-38 (deep red) and CCD-8Lu (red) fibroblasts or WI-38 (deep red) and LL97a (red) fibroblasts, isolated MT^GFP^ were located more abundantly in WI-38 fibroblasts than in CCD-8Lu or LL97a fibroblasts (Figure 2b). Similar MT^GFP^ transfer patterns were observed in cocultures of WI-38 (red) and CCD-8Lu (deep red) fibroblasts or WI-38 (red) and LL97a (deep red) fibroblasts (Figure S2b) regardless of the dye used. These results suggest that the transfer of mitochondria occurred preferentially according to fibroblast type in the order WI-38, LL97A, and CCD-8Lu fibroblasts, indicating that mitochondrial trafficking to specific cells took place.

To further investigate mitochondrial functionality in fibroblasts in the oxidative state, the oxygen consumption rate (OCR) was evaluated using an XF94 Flux analyzer after sequential injections of oligomycin, carbonyl cyanide 4-trifluoromethoxy-phenylhydrazone (FCCP), and antimycin A. CCD-8Lu fibroblasts exhibited the highest basal respiration, ATP-linked OCR, and maximal respiration, whereas the overall OCR was the lowest in WI-38 fibroblasts; thus, total oxidative capacity decreased in fibroblasts in the following order: CCD-8Lu, LL97A, and WI-38 (Figure 2c). Taken together, these data imply that the tendency of mitochondria to transfer to a specific cell might be determined by the quality of the mitochondrial function of that cell.

### 2.3. Preferential Transplantation of MT^GFP^ to Human Lung Fibroblasts with Mitochondrial Damage

**Figure 3:**
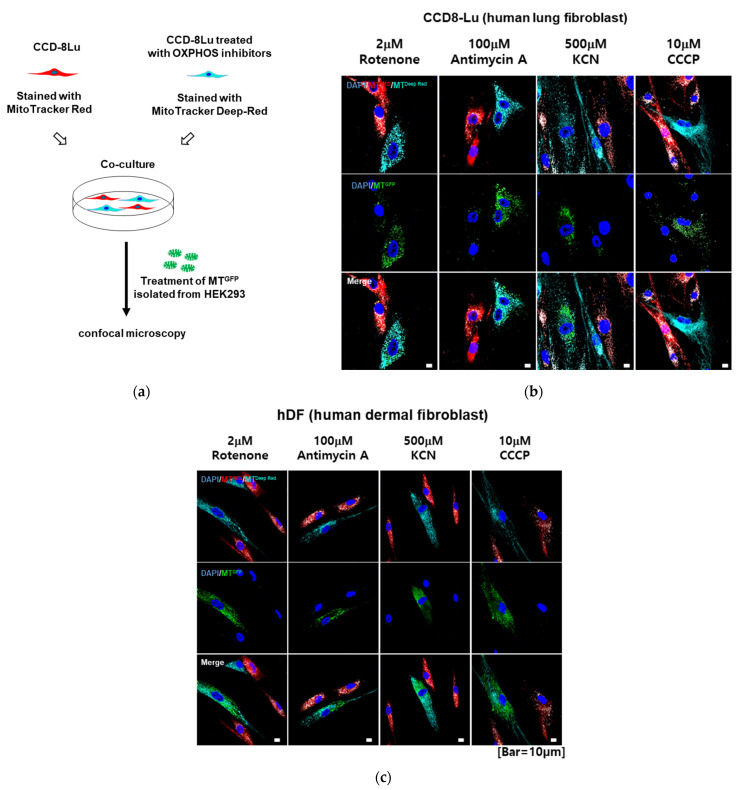
Preferential transplantation of MT^GFP^ to human lung fibroblasts with mitochondria damage. (**a**) Coculture model of normal fibroblasts and fibroblasts with OXPHOS inhibitor-induced artificial mitochondria damage. These two fibroblast groups were prelabeled with MitoTracker Red CMXRos and MitoTracker Deep-Red, respectively, cocultured and treated with MT^GFP^. MT^GFP^ was preferentially taken up by human lung fibroblasts (**b**) and human dermal fibroblasts (**c**) with damaged mitochondria. Cell nuclei were stained with DAPI (blue). Scale bar: 10 μm.

To ascertain whether the selective transfer of mitochondria was determined by the mitochondrial function of the receiving cells, we artificially induced mitochondrial damage in CCD-8Lu fibroblasts via treatment with OXPHOS inhibitors, including rotenone, antimycin A, potassium cyanide (KCN), and carbonyl cyanide m-chlorophenyl hydrazone (CCCP). As expected, intracellular ATP and mitochondrial membrane potential (MMP) were lower in OXPHOS inhibitors-treated CCD-8Lu cells than in untreated cells, whereas intracellular ROS were higher, indicating that the mitochondrial function of CCD-8Lu was significantly damaged by OXPHOS inhibitors (Figure S3). Untreated CCD-8Lu fibroblasts were prestained with MitoTracker Red CMXRos, and CCD-8Lu fibroblasts treated with OXPHOS inhibitors were prelabeled with MitoTracker Deep-Red. CCD-8Lu fibroblasts (red) and CCD-8Lu fibroblasts treated with OXPHOS inhibitors (deep red) were cocultured, treated with MT^GFP^ for 6 h, and observed under a confocal microscope (Figure 3a). Interestingly, more MT^GFP^ were found in rotenone-, oligomycin-, KCN-, and CCCP-treated CCD-8Lu fibroblasts than in untreated CCD-8Lu fibroblasts (Figure 3b). Similar uptake patterns were observed in cocultures of human dermal fibroblasts treated with OXPHOS inhibitors and untreated human dermal fibroblasts (Figure 3c). These data suggest that the selective migration of exogenous mitochondria to tissues or cells with damaged mitochondria occurred.

### 2.4. MT^GFP^ Trafficking to the Lung Tissue of BLM-Induced IPF Mice

**Figure 4:**
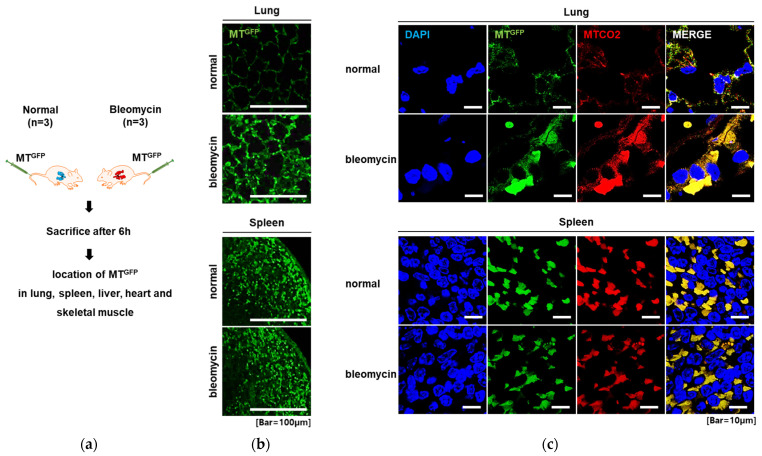
MTGFP trafficking to the lung tissue of BLM-induced IPF mice. (**a**) Schematic illustrating the experimental approach. (**b**) In vivo tracking of MTGFP in the lung and spleen. Normal and BLM-induced IPF mice were administered 10 μg of MTGFP intravenously. BLM-induced IPF mice showed stronger fluorescent signals in the lung than those of normal mice, whereas similar signals were detected in the spleens of these mice. Scale bar: 100 μm. (**c**) In the lung and spleen tissues of mice, MTGFP (green) were colocalized with MT-CO2 (red), which was detected using a human-specific anti-MT-CO2 antibody. Cell nuclei were stained with DAPI (blue). Scale bar: 10 μm.

To investigate the in vivo biodistribution of MT^GFP^, we tracked MT^GFP^ in the major organs of mice after they were injected systemically. In addition, we determined whether MT^GFP^ were enriched in the organs with mitochondrial damage. Because BLM induces mitochondrial damage [39,40] and DNA strand breaks, which cause pulmonary injuries and subsequent pulmonary fibrosis, BLM-induced IPF mice were used as a model to investigate the in vivo trafficking of MT^GFP^ (Figure 4a). After 6 h of intravenous injection of MT^GFP^, the major organs of mice were harvested, sectioned, and stained with an anti-GFP antibody. Fluorescence analysis via confocal microscopy revealed that GFP-labeled mitochondria were located in the spleen, skeletal muscle, lungs, liver, and heart in both normal mice and BLM-treated mice. Most organs showed similar fluorescent intensities in normal and BLM-treated mice (Figure 4b and Figure S4a); however, fluorescent intensity in the lung tissue was significantly higher in BLM-treated mice than in normal mice, suggesting that MT^GFP^ preferentially migrated to the tissue subjected to BLM-induced IPF. To verify the distribution of MT^GFP^, tissue sections were stained with an anti-MT-CO2 antibody that detects human-specific mitochondria. GFP was confirmed to be colocalized with human MT-CO2 in all tissues, indicating the presence of exogenous MT^GFP^ (Figure 4c and Figure S4b). Furthermore, MT-CO2 and MT^GFP^ were more enriched in the lung tissues of BLM-treated mice than in those of normal mice. Overall, these in vivo results corroborate the in vitro cell model results, i.e., mitochondrial trafficking occurred toward cells and tissues with mitochondrial damage both in vitro and in vivo. In the past decade, the ability of mesenchymal stem cells (MSCs) to migrate and engraft into sites of damage or inflammation has been reported [28,29,30,31,32]. For example, when MSCs were administrated intravenously into a mouse model of lung fibrosis, they preferentially migrated into the injured lung, resulting in beneficial outcomes, including inflammation reduction and collagen deposition [28]. Specific molecular interactions between chemokine receptors and their ligands expressed by the damaged tissue and MSCs are known to be critical in the trafficking of MSCs to their target destination [41,42]. The in vitro and in vivo results of the present study demonstrate for the first time that exogenous mitochondria preferentially traffic to and are taken up by cells and tissues wherein mitochondria are damaged. The molecular mechanism underlying the selective migration of mitochondria to tissues and cells with damaged mitochondria remains unknown. Further studies that reveal the molecular cues that steer the migration of mitochondria toward a target tissue may lead to the development of new treatments for the selective delivery of therapeutic agents to injured or diseased sites.

## 3. Materials and Methods

### 3.1. Cell Culture and Treatments 

Human dermal fibroblasts, human lung fibroblasts, and human embryonic kidney HEK293 cells were cultured in Dulbecco’s modified Eagle’s medium (DMEM; Welgene, Republic of Korea) supplemented with 10% fetal bovine serum and antibiotics (100 units/mL of penicillin and 100 μg/mL of streptomycin).

### 3.2. Plasmid DNA Preparation

The human TOM20 gene was fused with the GFP gene. First, human TOM20 was amplified via RT-PCR from a human cDNA library using forward (5′-AAA AAA CTC GAG CTA TGG GTC GGA ACA GCG CCA TCG-3′) and reverse (5′-AAA AAA GTC GAC TGT TCC ACA ATC TTC AGC CAA GC-3′) oligonucleotides. Amplified PCR products were cut with the restriction enzymes Xho I and Sal I and cloned into the plasmid pEGFP-N1, resulting in the plasmid pCMV_TOM20_GFP.

### 3.3. Generation of a Stable HEK293 Clone Expressing TOM20_GFP

Plasmid pCMV_TOM20_GFP was transfected into HEK293 cells. For transfection, the cells were plated in 6-well plates at 5 × 10^5^ cells/well in DMEM without antibiotics one day prior to transfection. Transfections were performed using Lipofectamine Plus (ThermoFisher Scientific, MA, USA) according to the manufacturer’s protocol. Stable transfectants were screened using geneticin (G-418; ThermoFisher Scientific, MA, USA) treatment. Specifically, the transfected cells were treated with geneticin (400 μg/mL), and TOM20_GFP stable transfected cell lines were screened after 5–10 days as follows: single stable clones were cultured confluently in 24-well plates and washed twice with 1× phosphate-buffered saline (PBS), lysed with 100 μL of cell lysis buffer for 15 min on ice, and then centrifuged for 15 min at 12,000× *g* to prepare the TOM20_GFP solution. This protein solution was then transferred into a new tube and mixed with an equal volume of SDS lysis buffer. Finally, the mixture was heated in boiling water for 5 min and subjected to western blotting analysis. 

### 3.4. Western Blot Assay

Proteins were subjected to 10% SDS-PAGE and transferred onto a nitrocellulose membrane. For blocking, the membrane was kept in 5% skimmed milk at room temperature for 1 h. After blocking, the membrane was incubated with rabbit polyclonal anti-GFP (A-6455, Invitrogen; 1:1000), mouse monoclonal anti-COX IV (ab33985, Abcam; 1:1000), mouse monoclonal anti-tubulin (BT7R, ThermoFisher Scientific, MA, USA; 1:5000), or mouse monoclonal anti-actin (A2228, Sigma, MO, USA; 1:5000) at 4 °C for 12 h with gentle shaking. The membrane was then washed three times with 1× TBST and incubated with goat anti-rabbit IgG HRP as a secondary antibody at room temperature for 1 h. Subsequently, the membrane was washed three times with 1× TBST, and images of protein expression were captured using an enhanced-chemiluminescent substrate (Amersham).

### 3.5. Isolation of Mitochondria from a TOM20_GFP-Producing HEK293 Clone

Cells were harvested from culture flasks, depressurized in SHE buffer [0.25 M sucrose, 20 mM HEPES (pH 7.4), 2 mM EGTA, and 0.1% defatted bovine serum albumin (BSA)] using nitrogen cavitation (Parr Instrument Co., IL, USA) [43] and then centrifuged at 2000× *g* and 4 °C for 10 min to remove cellular debris and nuclei. The supernatant was then centrifuged at 12,000× *g* and 4 °C for 15 min to pellet the mitochondria. The pellet was washed twice via suspension in 500 μL of SHE buffer, followed by centrifugation at 20,000× *g* and 4 °C for 10 min. The final pellet was resuspended in 100 μL of suspending buffer and kept on ice until use. Isolated mitochondria were quantified by determining protein concentrations using a bicinchoninic acid assay.

### 3.6. Mitochondrial Transfer in a Coculture System 

To discriminate the different cell types in a coculture system, monocultures were stained with MitoTracker Red CMXRos (ThermoFisher Scientific) or MitoTracker Deep Red (ThermoFisher Scientific). All staining procedures were performed using adherently growing cells in 100 mm tissue flasks. For staining, each cell was washed with 1× PBS twice and incubated for 30 min with MitoTracker Red CMXRos or MitoTracker Deep Red. After the staining solution was removed, the cells were washed with PBS three times, detached from the culture flasks via trypsinization, and quantified using a Neubauer hemocytometer. An equal density (5 × 10^3^ cells) of each stained cell type was coplated in a 24-well tissue plate and maintained at 37 °C for one day. The cells were then treated with 1 μg of mitochondria isolated from HEK293 cells stably expressing TOM20_eGFP. After incubation for 6 h, the cells were washed with 1× PBS twice, fixed in 4% formaldehyde, mounted in VECTASHIELD with DAPI mounting solution (Vector Laboratories Inc., Burlingame, CA, USA), and photographed using a confocal microscope (Olympus). 

### 3.7. Immunocytochemistry and Immunohistochemistry

Cells were fixed in 4% formaldehyde for 30 min, washed with 1× PBS twice, and then incubated with blocking buffer (PBS containing 10% goat serum and 0.2% Triton X-100) for 2 h. The cells were then incubated overnight at 4 °C with a primary antibody (rabbit polyclonal anti-GFP antibody; 1:1000) in PBS containing 1% normal goat serum and 0.1% Triton X-100. Following washes with PBS, appropriate fluorescence-tagged secondary antibodies (Jackson Immunoresearch Laboratories, West Grove, PA, USA) were used to achieve visualization. Stained samples were mounted in VECTASHIELD with DAPI mounting solution and photographed using the Olympus confocal microscope.

### 3.8. Measurement of the OCR

The OCR was analyzed in three types of lung fibroblast grown with an XF96 Extracellular Flux Analyzer (Seahorse Bioscience). Each type of fibroblast was seeded in an XF 96-well cell culture microplate (Seahorse Bioscience) at a density of 2 × 10^4^ cells/well in 250 μL of culture media and grown for 24 h at 37 °C. After substituting 590 μL of bicarbonate-free DMEM for the culture media, the cells were preincubated for 1 h. A baseline OCR measurement was collected, and the OCR was measured by adding 1 μM oligomycin, 0.3 μM FCCP, and 0.1 μM rotenone sequentially. The OCR was calculated in 3 min measurement cycles. From the OCR profiles, ATP-linked respiration (basal OCR–oligomycin-OCR) and maximal respiratory capacity (FCCP-OCR–rotenone-OCR) were calculated. Data were expressed as pmol of O2 per min and normalized according to cell number.

### 3.9. Intracellular ATP, Intracellular ROS, and Membrane Potential

CCD8-Lu fibroblasts were treated with 2 μM rotenone, 100 μM antimycin A, 500 mM KCN, and 10 mM CCCP overnight. Intracellular ATP was measured using a CellTiter-Glo Luminescence Kit (Promega, Madison, WI, USA). The cells were added to 96-well white-bottomed plates, and the CellTiter-Glo Luminescence reagent was added. The plates were placed on a shaker for 2 min and incubated for 10 min. The luminescent signals were evaluated using a microplate reader (Synergy HTX, Biotek, CA, USA). Estimates of intracellular ROS and membrane potential were performed via flow cytometry. ROS levels were detected using CM-H2DCFDA (Invitrogen) in cells. The fluorescent signal was emitted at 519 nm when H2DCFDA was transformed into fluorescent 2′,7′-dichlorofluorescein via ROS. The cells were incubated with 5 μM CM-H2DCFDA at 37 °C for 30 min and washed with Dulbecco’s phosphate-buffered saline (DPBS) twice before measurements were taken. The membrane potential was detected using a fluorescent probe of mitochondrial membrane potential, namely tetramethylrhodamine (TMRE) (Invitrogen). The cells were incubated with 500 nM TMRE at room temperature for 10 min and washed with DPBS twice before measurement. 

### 3.10. BLM-Induced IPF Mouse Model and the Injection of Mitochondria

Specific pathogen-free C57BL/6 male mice (7 weeks old) were purchased from Orient Bio (Seongnam, Republic of Korea) and used after acclimatization for one week. The animal facility was maintained under controlled conditions (relative humidity: 50% ± 20%; temperature: 23 °C ± 3 °C; light/dark cycle: 12/12 h) with 10–20 air changes per hour. The animals were fed commercial rodent chow (PMI Nutritional International Inc., Richmond, IN, USA) and filtered tap water ad libitum. All animal studies were approved by the Institutional Animal Care and Use Committee of the Korea Institute of Toxicology (Jeongeup, Republic of Korea; IACUC #2105-0014). To induce fibrosis in the lung parenchyma, the mice were anesthetized with inhaled isoflurane (Hana Pharm Co., Ltd., Hwa-Sung, Republic of Korea) and 1.8 mg/kg (36 μg/50 μL of saline/head) of BLM was administered via a single intratracheal instillation using an automatic video instillator [44,45]. The normal mice received an equivalent volume of saline. Healthy male mice (*n* = 3) and BLM-induced IPF mice (*n* = 3) were administrated intravenously with 20 μg of MTGFP eight days after BLM was administered to the animals. All mice were sacrificed 6 h after the MTGFP treatment. The spleen, thymus, liver, heart, lungs, and skeletal muscle were obtained during necropsy. These tissues were routinely fixed with 10% neutral buffered formalin solution and embedded in paraffin, after which they were sectioned to a thickness of 3–5 μm. The in vivo studies were performed in a Good Laboratory Practice setting.

### 3.11. Statistical Analysis

All statistical analyzes were performed using GraphPad Prism 5.03 (GraphPad Software, Inc., CA, USA). One-way and two-way ANOVA were used for multiple comparisons, and statistical significance was set at * *p* < 0.05, ** *p* < 0.01, and *** *p* < 0.005.

## 4. Conclusions

Mitochondrial transplantation therapy has emerged as one of the important therapeutic options in the management of diseases induced by mitochondrial dysfunction. To succeed the mitochondrial transplantation therapy, it is necessary for systemically administrated mitochondria to be able to migrate the sites with mitochondrial damage. Our in vitro and in vivo data clearly demonstrated that exogenous mitochondria are preferentially trafficked into and are subsequentially internalized to cells and tissues where mitochondria are damaged, suggesting that mitochondria transplantation can be an effective therapeutic strategy for treating mitochondrial dysfunction. Alternatively, mitochondria can be used as a tool for the delivery of therapeutic agents to injury sites.

## Data Availability

Data are contained within the article and Supplementary Materials.

## Supplementary Materials

The following supporting information can be downloaded at: https://www.mdpi.com/article/10.3390/ijms232415734/s1.
