# Supplementary material for: Preferred Migration of Mitochondria toward Cells and Tissues with Mitochondrial Damage
Source: Int J Mol Sci. 2022 Dec 12;23(24):15734. doi: 10.3390/ijms232415734 (PMC9779580; doi:10.3390/ijms232415734)
Supplement: Supplementary file 1 [file ijms-23-15734-s001.zip › ijms-2083928-supplementary.pdf]

Supplementary Materials

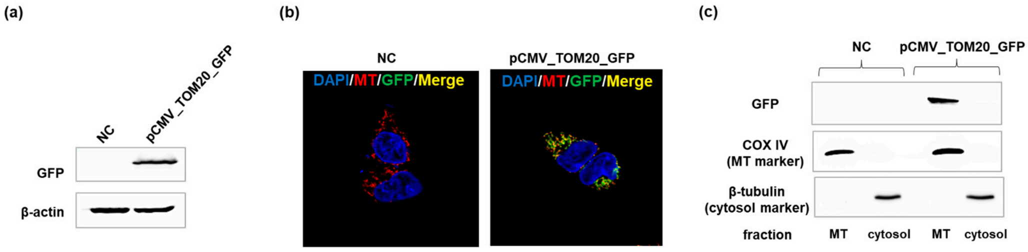

Figure S1. MTGFP trafficking to the lung tissue of BLM-induced IPF mice. (**a**) TOM20_eGFP protein was confirmed in HEK293 cells using a western blot assay. (**b**) Immunocytochemistry analysis revealed that eGFP protein was in HEK293 cells. The endogenous mitochondria of HEK293 cells were stained with MitoTracker Red CMXRos, and the cell nuclei were stained with DAPI. Scale bar: 10 μm. (**c**) Western blotting showed that the expression of eGFP was exclusively localized in the mitochondria. Anti-COX IV and anti- -tubulin antibodies were used as mitochondrial and cytosol markers, respectively.

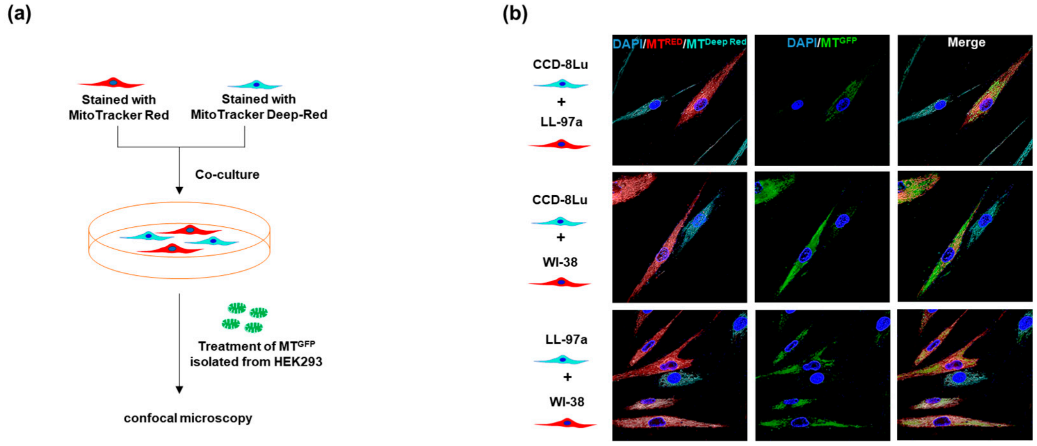

Figure S2. (**a**) Schematic of the coculture system. Each fibroblast type was prestained with MitoTracker Red CMXRos or MitoTracker Deep-Red. An equal density of each stained cell type was coplated, treated with MTGFP, and imaged using a confocal microscope. (**b**) Selective transplantation of MTGFP in the coculture system. LL-97a and WI-38 fibroblasts prestained with MitoTracker Red CMXRos took up more MTGFP than were taken up by CCD-8Lu fibroblasts prelabeled with MitoTracker Deep-Red (top and middle panels). Fluorescent images showed that MTGFP was more abundant in WI-38 fibroblasts (red) than in LL97a fibroblasts (deep red). Nuclear staining is shown in blue (DAPI). Scale bars: 10 μm.

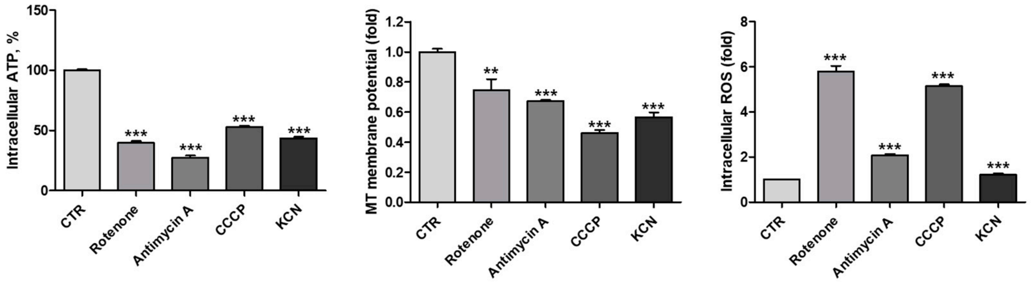

Figure S3. Effects of artificially induced mitochondrial damage via OXPHOS inhibitors in human CCD-8Lu fibroblasts. CCD-8Lu fibroblasts were treated with 2 μM rotenone, 100 μM antimycin A, 10 μM CCCP, and 500 μM KCN, respectively. Intracellular ATP content, membrane potential, and intracellular ROS level were determined to assess the effects of various OXPHOS inhibitors.

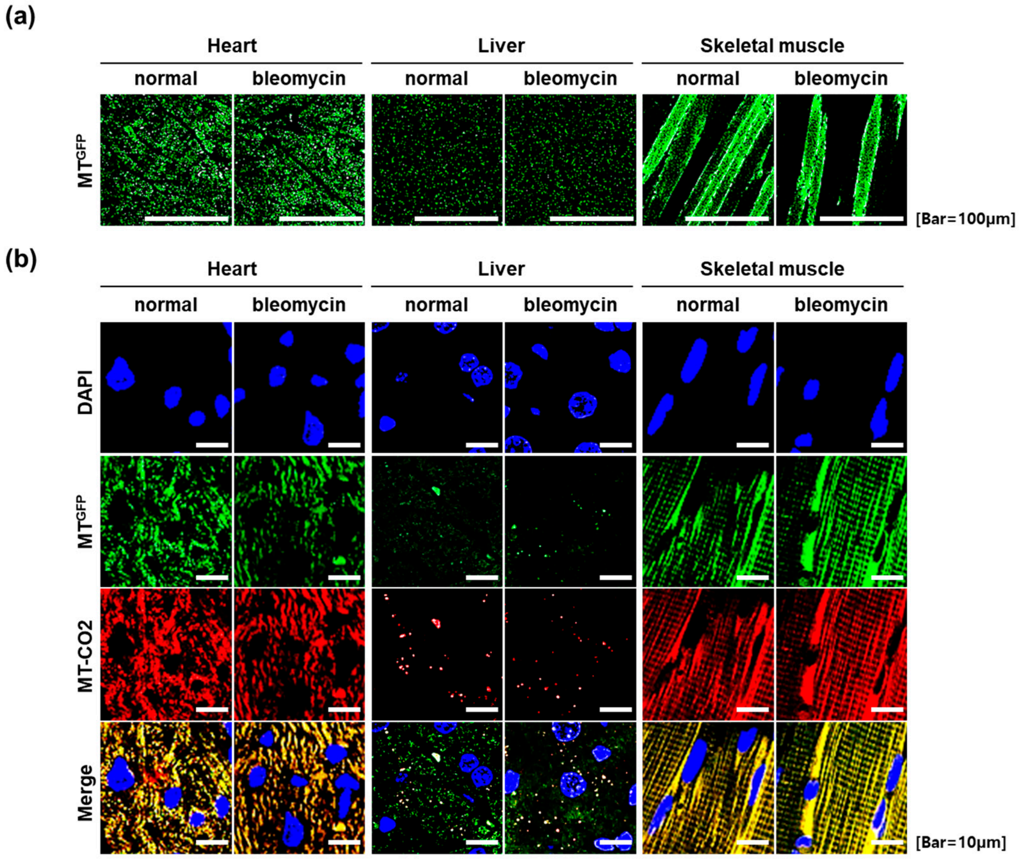

Figure S4. Assessment of MTGFP in mouse tissues, and colocalization of MTGFP with MT-CO2. (**a**) In vivo tracking of MTGFP in the heart, liver, and skeletal muscle of mice. Normal and BLM-induced IPF mice were administered 10 μg of MTGFP intravenously. No significant difference in GFP intensity was detected between the normal and BLM-induced IPF mice. Scale bar: 100 μm. (**b**) MTGFP (green) were colocalized with MT-CO2 (red), which was detected using human-specific anti-MT-CO2 antibody. Cell nuclei were stained with DAPI (blue). Scale bar: 10 μm.
